# Occurrence of OsHV-1 in *Crassostrea gigas* Cultured in Ireland during an Exceptionally Warm Summer. Selection of Less Susceptible Oysters

**DOI:** 10.3389/fphys.2016.00492

**Published:** 2016-11-08

**Authors:** Maria Prado-Alvarez, Grainne Darmody, Stephen Hutton, Amy O'Reilly, Sharon A. Lynch, Sarah C. Culloty

**Affiliations:** Aquaculture and Fisheries Development Centre, School of Biological, Earth and Environmental Science and Environmental Research Institute, University College CorkCork, Ireland

**Keywords:** *Crassostrea gigas*, OsHV-1μvar, prevalence, resistance, qPCR, ISH

## Abstract

The occurrence of OsHV-1, a herpes virus causing mass mortality in the Pacific oyster *Crassostrea gigas* was investigated with the aim to select individuals with different susceptibility to the infection. Naïve spat transferred to infected areas and juveniles currently being grown at those sites were analyzed using molecular and histology approaches. The survey period distinguishes itself by very warm temperatures reaching up to 3.5°C above the average. The virus was not detected in the virus free area although a spread of the disease could be expected due to high temperatures. Overall mortality, prevalence of infection and viral load was higher in spat confirming the higher susceptibility in early life stages. OsHV-1 and oyster mortality were detected in naïve spat after 15 days of cohabitation with infected animals. Although, infection was associated with mortality in spat, the high seawater temperatures could also be the direct cause of mortality at the warmest site. One stock of juveniles suffered an event of abnormal mortality that was significantly associated with OsHV-1 infection. Those animals were infected with a previously undescribed microvariant whereas the other stocks were infected with OsHV-1 μVar. Cell lesions due to the infection were observed by histology and true infections were corroborated by *in situ* hybridization. Survivors from the natural outbreak were exposed to OsHV-1 μVar by intramuscular injection and were compared to naïve animals. The survival rate in previously exposed animals was significantly higher than in naïve oysters. Results derived from this study allowed the selection of animals that might possess interesting characteristics for future analysis on OsHV-1 resistance.

## Introduction

*Crassostrea gigas* has become the oyster of choice for cultivation in many regions of the world due to its rapid growth and wide tolerance to environmental conditions. Although, mortality outbreaks of this species have been reported worldwide since the 1950's (Pereyra, 1964), the occurrence of a virus associated with mass mortalities was described in the early 90's in countries as apart as France and New Zealand (Hine et al., 1992; Nicolas et al., 1992; Renault and Cochennec, 1994). Virus isolated from larvae was referred to as ostreid herpesvirus-1 (OsHV-1), included into the Herpesviridae family and genetically characterized (Minson et al., 2000; Davison et al., 2005). OsHV-1 were consecutively reported in different countries confirming a global distribution (Renault and Arzul, 2001; Friedman et al., 2005; Moss et al., 2007; Garcia et al., 2011).

Compared to the reference type, the variant OsHV-1 Var is characterized by a deletion of 2.8 Kbp (Arzul et al., 2001) and the variant OsHV-1 μVar shows, among other polymorphisms, a 12 pb deletion in a microsatellite area in the C region (Segarra et al., 2010). Other microvariants described afterwards exhibited nucleotide mismatches in this area of the genome. OsHV-1 μvar and these microvariants were particularly virulent being detected in oyster batches that suffered mass mortalities (Segarra et al., 2010; Martenot et al., 2011; Lynch et al., 2012; Peeler et al., 2012; Pernet et al., 2012; Renault et al., 2012; Roque et al., 2012).

Among aspects influencing the development of the disease, a rapid increase in the sea water temperature seems to be a critical factor (EFSA, 2010; Garcia et al., 2011). Indeed, massive mortalities are not usually observed below 16°C (EFSA, 2010). In order to find a method to mitigate the disease in cultivable stocks, experimental movements of oysters at different temperatures have been recently tested (Petton et al., 2013, 2015; Pernet et al., 2015). However, preliminary results concluded that movements of oyster would only delay mortality and also increase the spread of the disease (Pernet et al., 2015).

Treatment against diseases is generally not feasible in bivalves due to the lack of an acquired immune response. Therefore, genetic selection and selective breeding programs should play an important function in increasing the productivity of aquaculture operations. Resistance to mortality was described as a highly heritable trait in oysters (Sauvage et al., 2010) and genetic selection of resistant animals seems to be the most plausible alternative to reduce oyster mortality in the field (Dégremont, 2013). Indeed, significant efforts are being carried out in different countries including Ireland (Dégremont, 2013; Clegg et al., 2014).

In the present study, the status of different stocks of spat and juveniles in three Irish farms was evaluated. Different diagnosis methods including molecular detection, histological observation and *in situ* hybridization were combined in this study for a better identification of OsHV-1 infected animals. Less and more susceptible animals were selected by their survival after a natural outbreak and after an experimental infection under laboratory conditions.

## Materials and methods

### Sampling of 1-year old *Crassostrea gigas*

**Figure 1:**
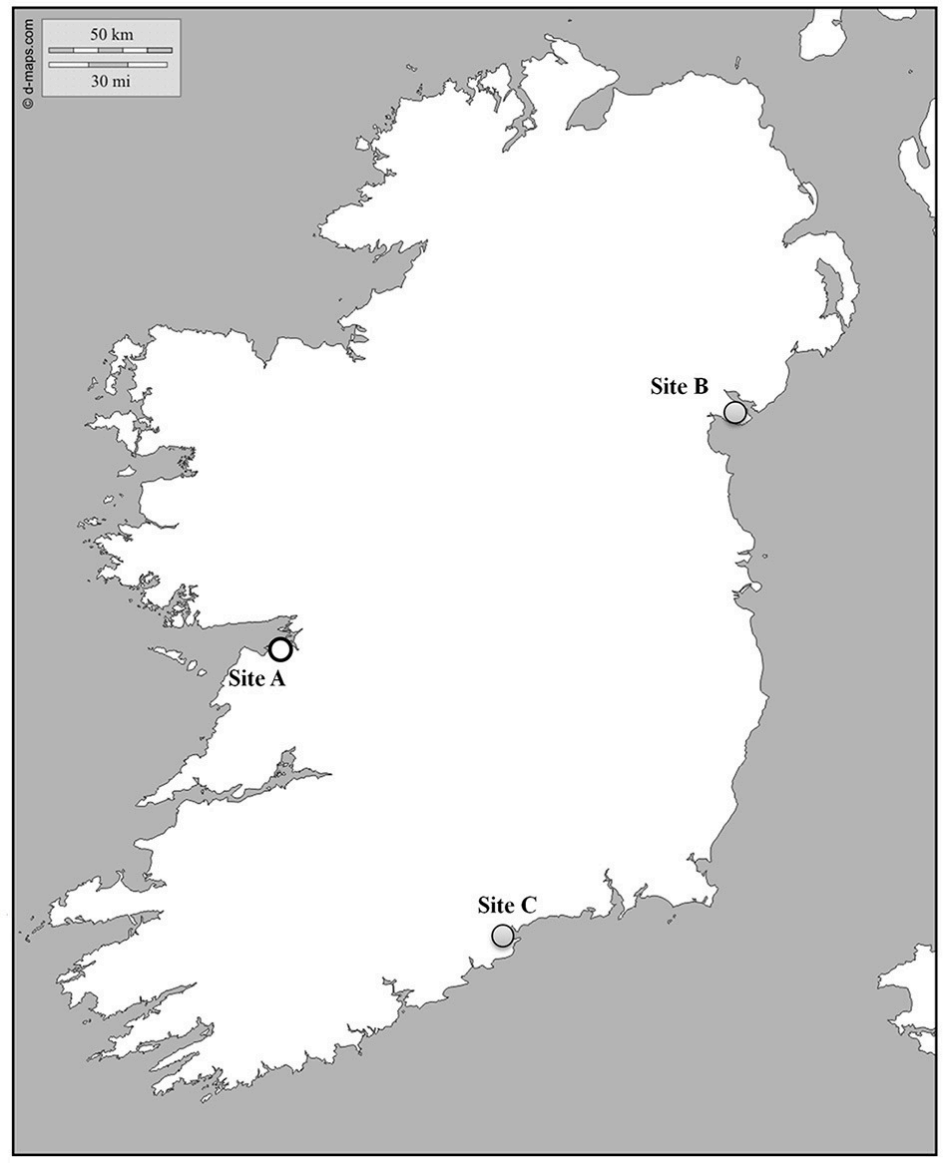
**Map showing the location of the sampling sites**. Gray and white dots indicate infected and virus free areas, respectively.

*Crassostrea gigas* oysters were screened from three different shellfish farms located along the Irish coast (Figure 1). Oysters were locally produced and grown at Site A or imported from an external hatchery and settled at Site B and Site C. Three consecutive bags per site, containing juveniles (12 months old, 6.9 ± 1.5 cm in length) were sampled every fortnight. Percentage of mortality was estimated in 100 oysters per bag by counting coupled empty shells. After removal of all dead animals, 20 oysters per bag were collected, transferred to the laboratory facilities in refrigerated boxes and processed within 24 h for tissue collection and histology.

### Experimental transfer of naïve Irish spat to OsHV-1 infected areas

An initial sample of 30 spat of 4-months old (1.02 ± 0.35 cm in length) produced in Ireland (Site A) was tested by standard PCR to confirm the absence of the virus before being transferred to two infected sites (Site B and C). Primer pairs OHVA and OHVB (Lynch et al., 2013) gave no amplification in any sample. 500 spat were settled by triplicates in bags in the same lines where juveniles were simultaneously sampled. Collection of oysters and the estimation of mortality were carried out as described above.

### Sea water temperature record

Temperature loggers (DST CTD, Star Oddi), placed in the same area where the oysters were sampled, recorded temperature data every hour. Maximum temperature reached per day was also included in the analysis.

### Animal processing and tissue collection

Shell length and wet weight were measured before dissection. A piece of gill tissue in juveniles was immediately collected after dissection and kept frozen (−20°C) until use. A cross section including the gill, mantle and gonad was fixed in Davidson's solution for 48 h and preserved in Ethanol (70%) before being processed for histology. Depending on the size of the animal and in order to obtain enough DNA material, whole organism excluding the digestive gland or a mix of gill and mantle were collected in spat oysters.

### DNA extraction

Genomic DNA was extracted from gills (juveniles) or mix of tissues (spat) using the DNeasy Blood and Tissue kit (Qiagen) following the manufacturer's instructions. DNA concentration and quality was assessed by spectrophotometer (NanoDrop, Thermo-Scientific). The number of samples to be analyzed per sampling point was decided attending to the percentage of mortality, 15 animals were processed when normal mortality was observed and 30 in the case of abnormal mortality. A total of 375 juveniles were processed for DNA extraction in five sampling times and 176 spat oysters collected in three sampling times.

### Oyster screening for OsHV-1 detection and sequencing

According to the method of Lynch et al. (2013) standard PCR using OHVA and OHVB primers (Table 1) was assayed in undiluted DNA samples to determine infection. A total of 105, 120, and 150 juveniles and 45, 90, and 41 spats were screened from Site A, B, and C, respectively. Prevalence of infection was estimated as the mean percentage of positive samples per oyster bag (*n* = 3). A selection of positive samples (40) was subsequently sequenced to identify the OsHV-1 microvariant. The amplicon obtained with C2-C6 primers (Renault and Arzul, 2001) (Table 1) was diluted in distilled water (1:10) and used as a template in a second round of PCR. Two combinations of primers were used to identify microvariants: (1) OHVC and OHVD primers (Lynch et al., 2013) giving a final fragment of 296 bp to amplify the microsatellite area and (2) C2-C2rev (Lynch et al., 2013) giving a fragment of 400 bp to identify the characteristic polymorphism of Irish OsHV-1 μvar variant. These sequences overlapped and were assembled to obtain a longer fragment for homology searches. PCR products were cleaned up using QIAquick gel extraction kit (Qiagen) and sequenced by the Sanger method using the corresponding primers pairs used for amplification, (SourceBioScience). Raw chromatograms were analyzed using Chromas 231 software (Technelysium). ExPaSy tools (http://us.expasy.org/genomics) and CAP3 (http://doua.prabi.fr/software/cap3) were used for sequence assembly. Multiple sequence alignment were executed by Muscle (Edgar, 2004) and visualized and edited using BioEdit v.7.2.5 (http://www.mbio.ncsu.edu/bioedit/page2.html). Searches of homology were performed on GenBank database using the Blast algorithm (http://ncbi.nlm.nih.gov/blast/). Sequences were deposited on GenBank with the accession number: KX147758.

**Table 1:** **List of primers used in this study**.

**Primer**	**Sequence (5′- 3′)**	**Amplicon**
C2-C6	CTCTTTACCATGAAGATACCCACC	709 bp
(Renault and Arzul, 2001)	GTGCACGGCTTACCATTTTT	
OHVA-OHVB	TGCTGGCTGATGTGATGGCTTTGG	385 bp
(Lynch et al., 2013)	GGATATGGAGCTGCGGCGCT	
OHVC-OHVD	AGGCGCGATTTGTCAGTTTAGAATCAT	296 bp
(Lynch et al., 2013)	AGGTTCAGGTCTTTGCGTTCCGT	
C2-C2rev	ATTGATGATGTGGATAATCTGTG	400 bp
(Lynch et al., 2013)	TTTGGTCAAGGTGCAAAATTC	
HVDPF-HVDPR	ATTGATGATGTGGATAATCTGTG	197 bp
(Webb et al., 2007)	GGTAAATACCATTGGTCTTGTTCC	

### OsHV-1 quantification by real-time PCR

The quantification of OsHV-1 was carried out by real-time PCR following the Standard Operating Procedure “OsHV-1 detection and quantification by Real Time Polymerase Chain Reaction using OsHV-1 DNA polymerase sequence” (http://www.eurl-mollusc.eu/SOPs) using the HVDP-F and HVDP-R primers (Webb et al., 2007) Each sample was analyzed in triplicate with 5 μl of genomic DNA (5 ng/μl) as template. The reaction contained 12.5 μl of Brilliant SYBR Green Mater Mix reagent (Agilent), 2.5 μl of HVDP-F (5 μM), 2.5 μl of HVDP-R (5 μM) (Table 1) and 2.5 μl of distilled water. Amplification was carried out in 384-microwell plates in an ABI 7900HT Real Time PCR system (Applied Biosystems). The standard cycling conditions consisted of an incubation at 95°C for 10 min allowing enzyme activation followed by 40 cycles of product melt at 95°C for 30 s, primer annealing at 60°C for 1 min and polymerase extension at 72°C for 45 s. A final melting curve analysis was developed following the instrument specifications. Negative controls containing distilled water were included on the plate and showed no amplification. The quantification of virus copies was extrapolated from a standard calibration curve prepared with serial dilutions of a known suspension of OsHV-1 genomic DNA (5 × 10^5^ copies/μL).

### Experimental infection with OsHV-1 μVar

Spat survivors from the natural outbreak were transferred to the laboratory facilities and let acclimatize before carrying out an experimental trial with a purified suspension of OsHV-1 μVar. 48 animals from each site (Site A and Site B) were settled in 4 tanks, 2 for virus injected animals and 2 for controls. Spat were injected in the adductor muscle with 50 μl of OsHV-1 μVar suspension (10^4^ DNA viral copies/μl) after being anesthetized using Magnesium Chloride (Suquet et al., 2009) and set up at constant temperature (22°C) under photoperiod regimen. Although, the number of infective particles was unknown, the suspension was previously tested to check infectivity. Control spat were injected with ultraviolet treated and 0.22 μm filtered sea water and kept under the same conditions. Mortality was recorded twice daily and dead animals were removed and screened for OsHV-1μvar detection and estimation of prevalence as described above.

### Histology and *in situ* hybridization

Fixed body sections of infected oysters were processed for histology and *in situ* hybridization (ISH). Tissues were dehydrated and embedded in paraffin wax. Blocks were cut in 7 μm sections. Standard histology procedures were carried out before staining with haematoxylin and eosin (Sigma) and covered using DPX mounting medium (Sigma). A selection of juveniles confirmed positive by PCR were processed for ISH to detect OsHV-1 in tissues. DNA digoxigenin-labeled probes were synthesized by PCR with OHVC- OHVD primers (Lynch et al., 2013) using the DIG Nucleid Acid Detection Kit (Roche). Solutions and buffers were freshly prepared following Sambrook and Russell (Sambrook and Russell, 2001). Procedure for hybridization, detection of DIG labeled DNA and counterstaining was made following Lynch et al. (2010) with minor modifications for OsHV-1 detection. Briefly, incubation of slides in moist chamber at 37°C for tissue preparation was reduced to 7 min, a higher volume of probe was used to detect OsHV-1 (10 μl) and incubation with nitroblue tetrazolium and 5-bromo-4-chloro-3-indolyl phosphate (NBT/BCIP) was extended to 2 h in the dark. Slides were observed and photographed under light microscopy at 40 × and 100 × magnification (Nikon Eclipse 80i).

### Statistics

Data were analyzed following t-Students Test. Chi square test was used to compare percentage of mortality, prevalence of infection and sea water temperature. Significant differences were considered at *p* ≤ 0.05. Data are presented as mean ± standard error. Statistical analyses on sea water temperature were carried out on BoxPlotR.

## Results

### Sea water temperature record in oyster bags

**Figure 2:**
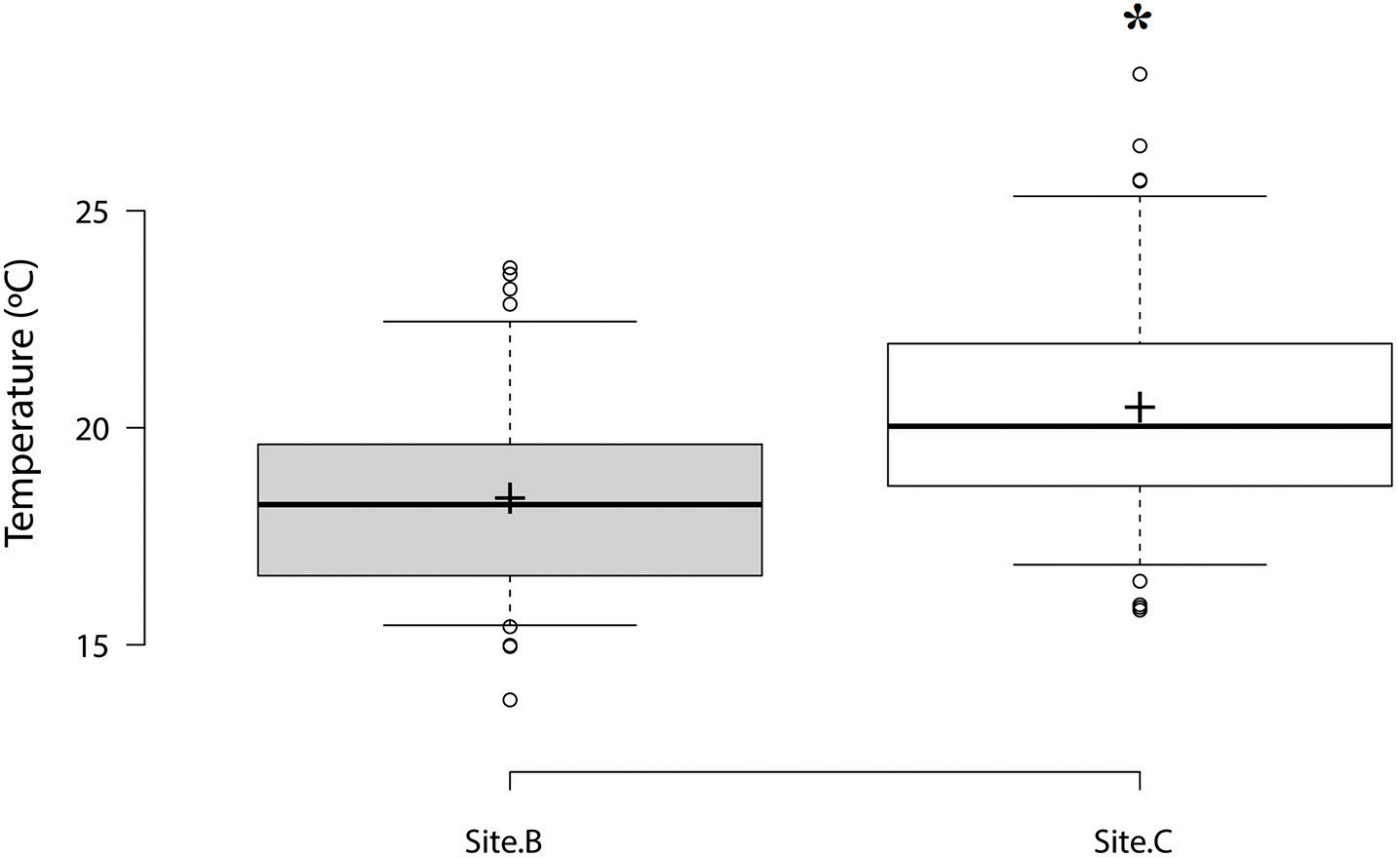
**Box plot of maximum temperatures per day at Site B and Site C**. Center lines show the median, box limits indicate the 1st and 3rd quartile. Sample means are represented by crosses. Significant differences are designated with an asterisk (*n* = 73, *p* ≤ 0.05).

The highest temperature per day varied between 13.7°C and 23.6°C at Site B with a mean value over the survey period of 18.3 ± 0.25°C. Mean temperature was significantly higher at Site C (20.4 ± 0.31°C), varying between 15.8°C and 28.1°C (Figure 2). Temperature varied by 3°C between 1st and 3rd quartiles for both sites. 75% of values recorded were below 19.6°C at Site B and below 21.9°C at Site C. Analysis of temperature per hour showed that oysters from Site C were exposed to 16°C or higher temperatures for longer than oysters from Site B.

### Prevalence of OsHV-1 and mortality in juveniles

**Figure 3:**
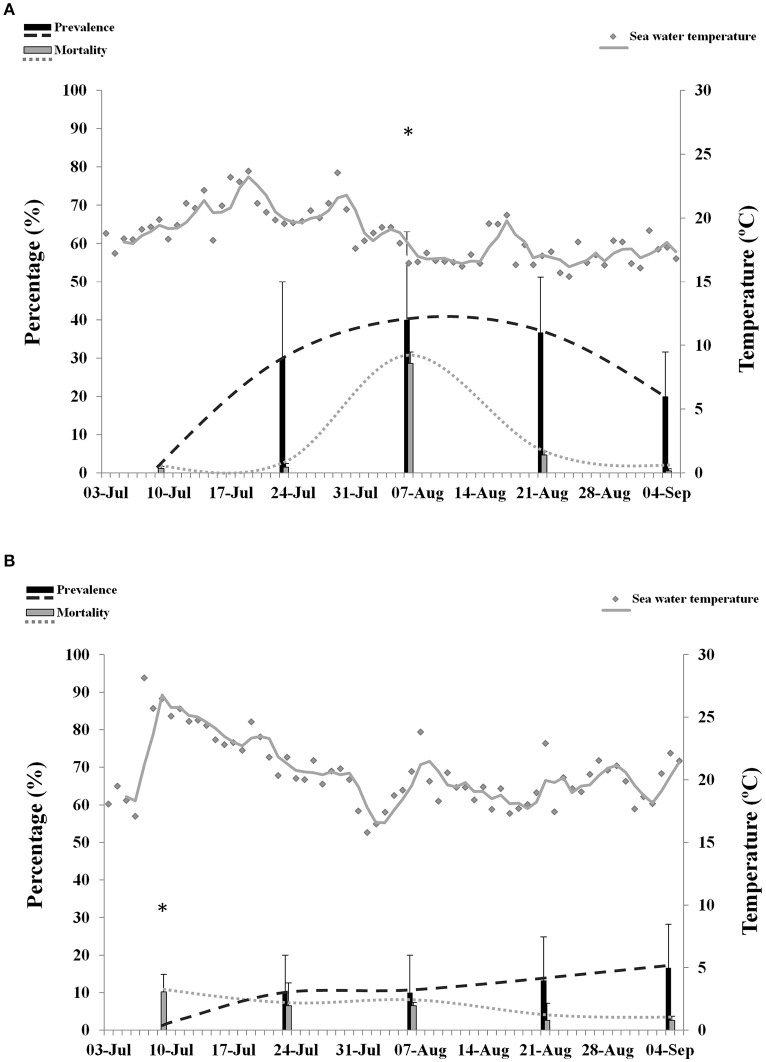
**Percentage of mortality and prevalence of OsHV-1 in juveniles collected from Site B (A)** and Site C **(B)**. Maximum sea water temperature on each site is represented with gray dots and gray trend line. Asterisks show significantly higher mortality (*n* = 3, *p* ≤ 0.05).

OsHV-1 was detected by PCR in juveniles from Site B (6.1 ± 0.8 cm) and Site C (5.8 ± 0.9 cm). OsHV-1 was not detected in naïve oysters from Site A (8.5 ± 0.9 cm) during the survey. OsHV-1 was detected in both infected areas from sampling 2 to sampling 5 (Figures 3A,B). Overall results showed significantly higher prevalence in juveniles from Site B (25%) compared to Site C (10%). Maximum prevalence of infection was 80% at Site B and 30% at Site C. No significant differences were observed on prevalence of infection per sampling point.

Maximum values of mortality reached 34.6% at Site B and 14.2% at Site C. Although, overall mortality was not significantly different between the two locations, comparison per sampling point showed significantly higher mortality at Site B (28.6%) compared to Site C (6.5%) at sampling point 3. No abnormal mortality was observed at Site A.

Episodes of high temperatures, considered in this study as a period of 14 consecutive days with highest maxima over 16°C, were not associated with mortality in juveniles in any of the infected sites. Prevalence of infection over 20% was significantly associated with abnormal mortalities at Site B (*p* = 0.03). However, a higher significance (*p* = 0.006) was obtained when temperature was included in the analysis. No significant correlation between mortality, temperature and prevalence was found at Site C.

### Prevalence of OsHV-1 and mortality in spat

**Figure 4:**
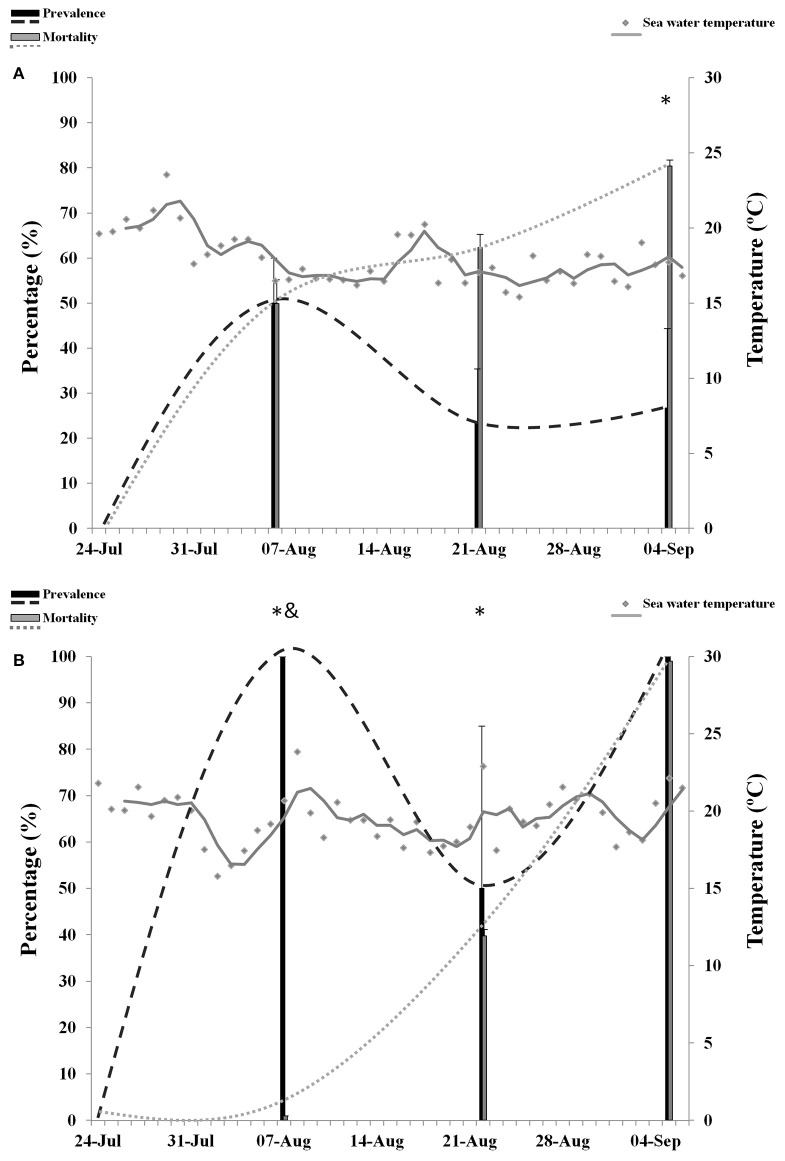
**Percentage of mortality and prevalence of OsHV-1 in spat collected from Site B (A)** and Site C **(B)**. Maximum sea water temperature on each site is represented with gray dots and gray trend line. Asterisks and ampersand show significantly higher mortality and prevalence, respectively (*n* = 3, *p* ≤ 0.05).

The overall size of spat transferred to Site B (1.19 ± 0.2 cm) was significantly lower than spat from Site C (1.4 ± 0.3 cm) and spat that remained at Site A (1.5 ± 0.5 cm). Prevalence of infection reached 70% at Site B (Figure 4A) and 100% at Site C (Figure 4B), whereas OsHV-1 was not detected in spat from Site A. No abnormal mortality was observed at Site A.

Overall mortality in spat was not significantly different between sites B (64.2%) and C (46.6%). Analysis per sampling point showed that mortality was significantly higher at Site B in samples 3 (49.9%) and 4 (62.3%). At sample 5, mortality increased significantly in spat from Site C. Overall prevalence of infection was significantly higher at Site C (83.3%) compared to Site B (33.3%). However, at sampling point 3 the prevalence of infection was higher at Site B and no significant differences were observed at sampling point 4 and 5. Abnormal mortality in spat was significantly related to the presence of OsHV-1 at both sites (*p* = 0.01, Site B; *p* = 0.04, Site C). Episodes of high temperatures were also related to mortalities at Site C (*p* = 0.04) but not at Site B (*p* = 0.2).

### Quantification of OsHV-1 in naturally infected samples

**Figure 5:**
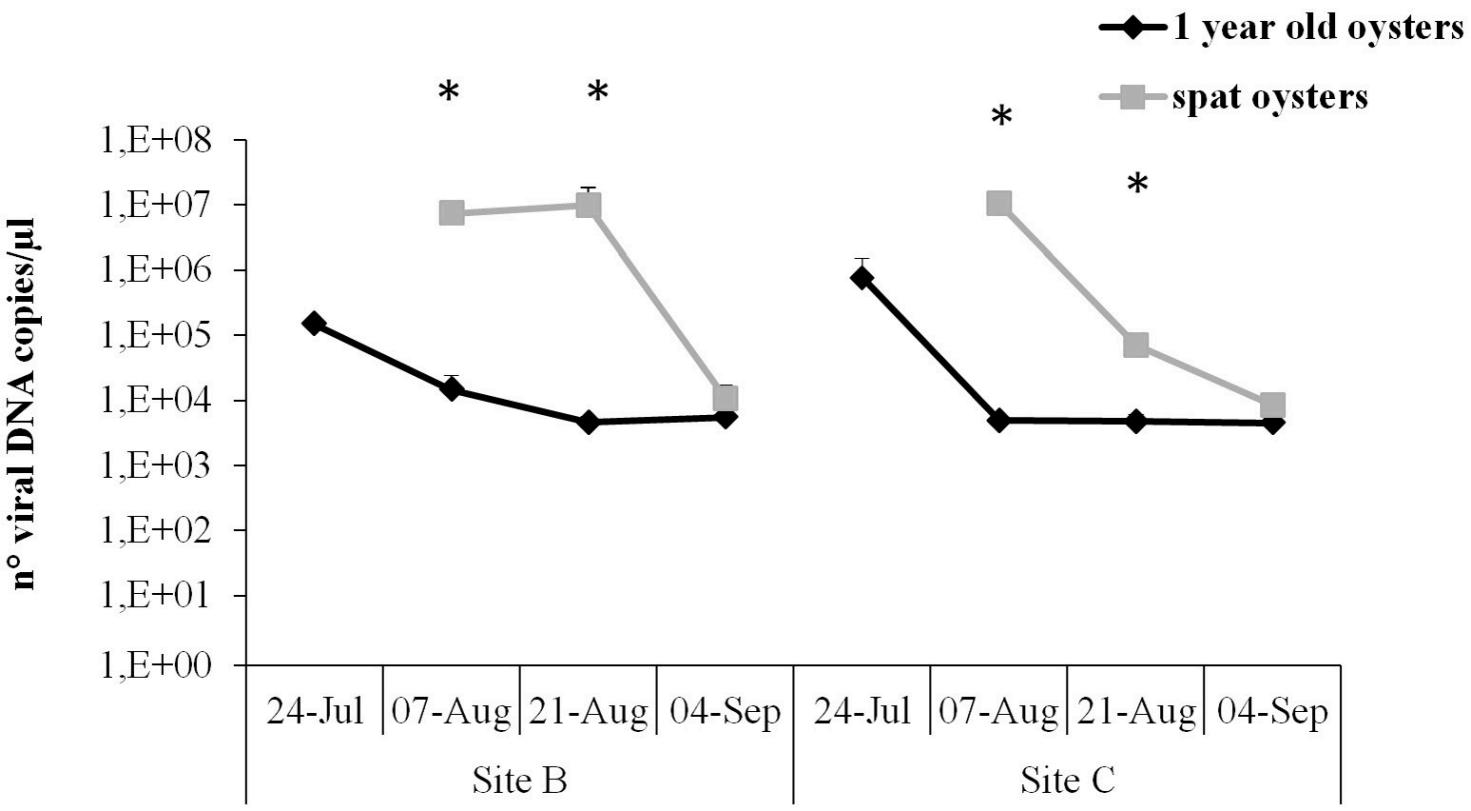
**Virus DNA quantification by real-time PCR in juveniles and spat from Site B and Site C at different sampling times**. Significant differences between oyster stages are indicated with asterisks (*n* = 6, *p* ≤ 0.05).

OsHV-1 was quantified by qPCR in a selection of samples that were positive by standard PCR. Overall viral load was significantly higher in spat (4.7 × 10^6^ DNA viral copies) compared to juveniles (1.35 × 10^5^ DNA viral copies) (Figure 5). No significant differences were observed between Sites for any of the oyster stages analyzed.

In juveniles from Site B and Site C and spat from Site C the highest viral load was observed at the beginning of the survey, reaching between 1.5 × 10^5^ and 1.07 × 10^7^ DNA viral copies. However, maximum viral load in spat from Site B was reached later, at sampling point 4. Number of viral copies was significantly higher in spat compared to juveniles in sampling point 3 and 4. The viral load decreased in all samples, showing no significant differences between samples and reaching a mean of 7.7 × 10^3^ viral copies at the end of the survey.

### Identification of OsHV-1 variants in naturally infected oysters

**Figure 6:**
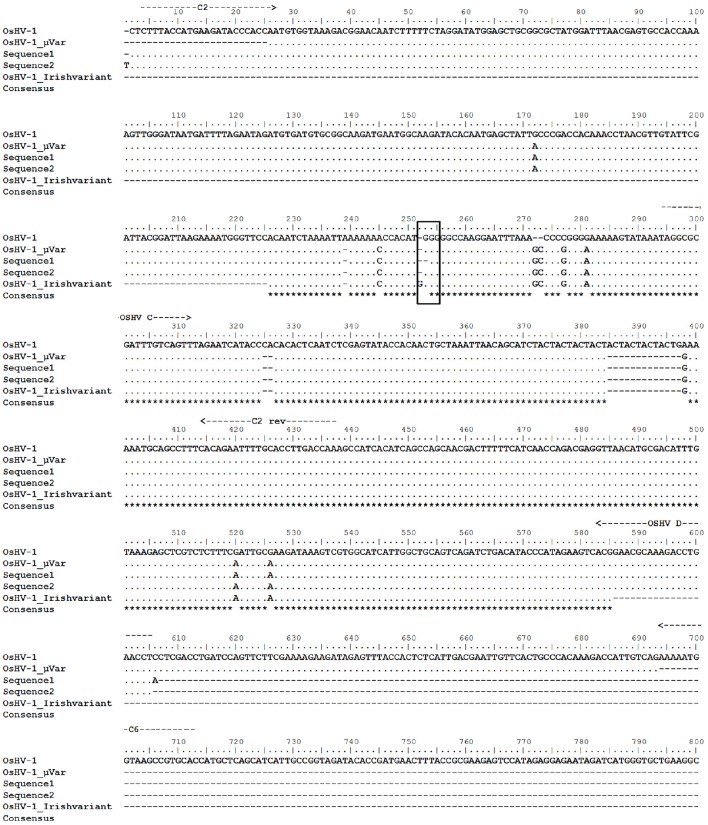
**Alignment of the nucleotide sequences obtained in this study (sequence 1 and sequence 2) with previously deposited sequences (OsHV-1 reference genome, acc num AY509253; OsHV-1 μVar, acc num HQ842610.1; Irish OsHV-1 μVar acc num JQ963169.1)**. Location of primers is indicated with arrows. Framed area indicates the location of the polymorphism found in sequence 2 compared to OsHV-1 μVar. Asterisks indicate conserved residues.

Positive amplicons obtained by the combination of different primer pairs were purified from the agarose gel using the QIAquick Gel Extraction Kit (Qiagen) and bidirectionally sequenced with specific primers. Chromatograms were carefully analyzed to discard any erroneous reading. All sequences from the same set of positive samples were identical. Multiple alignment grouped sequences in two main contigs obtaining two consensus sequences (Table 2). One contig containing 57 sequences from Site C juveniles and both spat stocks (sequence 1) and the other one containing only the 25 sequences from Site B juveniles (sequence 2). Nucleotide Blast searches revealed that sequence 1 had higher identity to OsHV-1 μVar than sequence 2 and both sequences had the same identity compared to the reference genome OsHV-1 (Table 2). Differences between consensus sequences described herein and previous microvariants sequences were observed in the nucleotide alignment (Figure 6). The polymorphisms on the C region that characterized the μVar variant were conserved in the two consensus sequences including the microsatellite deletion. One nucleotide mismatch was observed between sequence 1 and 2 (Figure 6, boxed region), consisting on a Guanine deletion at position 178430 (Davison et al., 2005). Compared to OsHV-1 μVar, translation of sequence 2 produced a different amino acid sequence at the beginning of ORF5. However, the protein sequence of ORF4 was not modified by this polymorphism (data not shown).

**Table 2:** **Consensus sequences generated by sequencing of positive samples and their percentage of identity to previous annotated genotypes based on BlastN local searches**.

		**Consensus sequence 1**	**Consensus sequence 2**
		**Spat: Site B and C**	**Juveniles: Site B**
		**Juveniles: Site C**	
**Identity**	OsHV 1 μvar	100%	99%
	(HQ842610.1)		
	OsHV-1	96%	96%
	(AY509253)		

### Spat survival after OsHV-1 μVar injection

**Figure 7:**
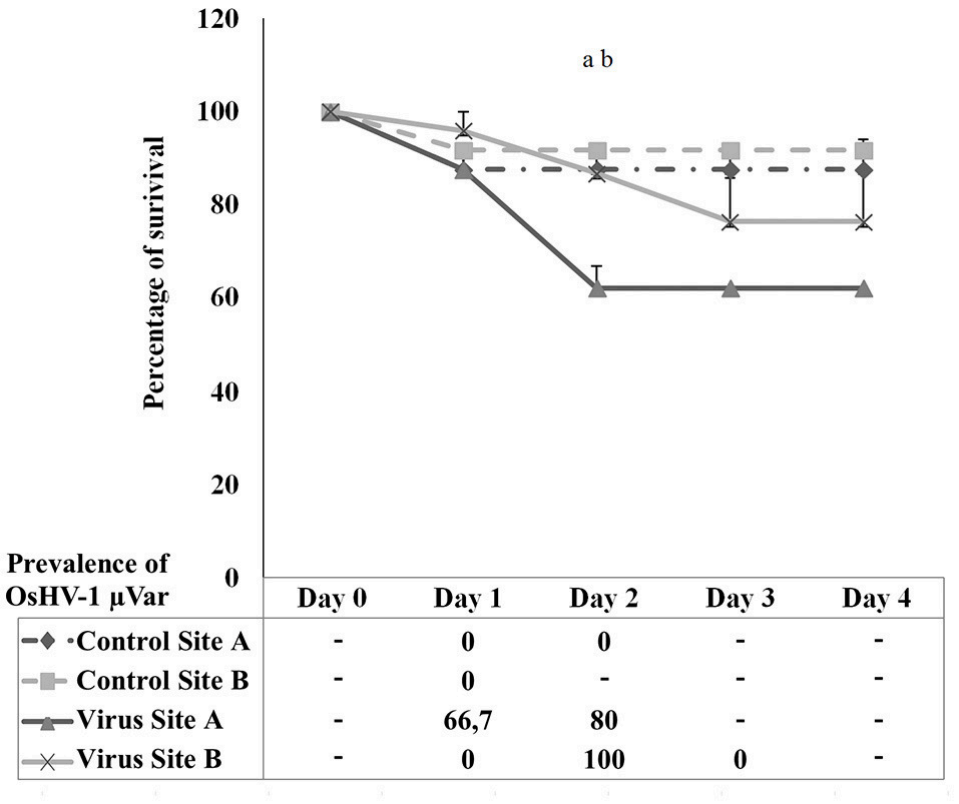
**Percentage of survival in spat oysters injected with OsHV-1 μVar (virus) and sea water (control) collected at Site A and Site B**. Table below the graph shows the prevalence of infection in dead animals collected at each sampling point. (a) Significant differences between control and virus at Site A. (b) Significant differences between Site A and Site B in virus exposed animals (*n* = 2, *p* ≤ 0.05).

Figure 7 shows the percentage of survival after intramuscular injection of OsHV-1 μVar in spat survivors from the natural outbreak. A decrease on survival was observed at 24 h post-injection in all injected animals. Dead animals from control conditions were not positive for OsHV-1 μVar detection by standard PCR over the 4 days trial. At 48 h post-injection, virus injected spat from Site A experienced a significant decrease in survival compared to controls (*p* = 0.02). No significant differences were observed between virus and control exposed animals from Site B (*p* = 0.05). Comparison between the two stocks revealed that survival was significantly higher in virus injected animals from Site B compared to virus injected animals from Site A (*p* = 0.03). Prevalence on virus injected animals was observed from day 1 in Site A and from day 2 in Site B reaching a peak of 80 and 100%, respectively.

### Histological examination of naturally infected animals and detection of OsHV-1 in tissues

**Figure 8:**
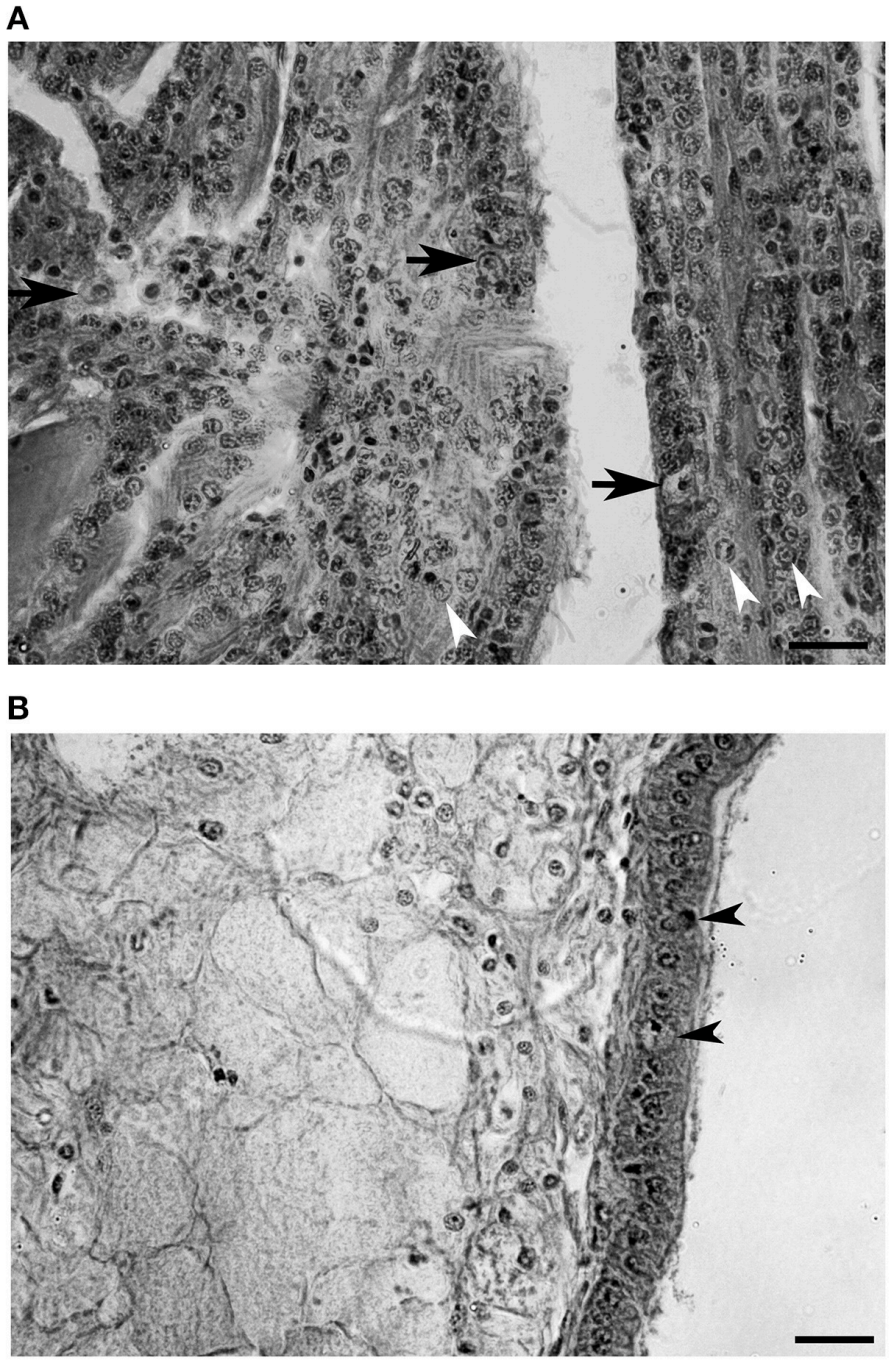
**Histological section of a heavily infected oyster. (A)** Gill tissue. Enlarged cells are indicated with arrows and chromatic margination is indicated with arrowheads. **(B)** Mantle tissue. Epithelial cells showing abnormal nucleus are indicated with arrowheads. (Scale bars, 20 μm).

Cellular changes were predominantly observed in gills (Figure 8A). Hypertrophied cells with a low nucleus-cytoplasm ratio (arrows, Figure 8A) and nuclear abnormalities such as chromatin fragmentation and margination, and pycnosis were observed (arrowheads, Figure 8B). Infiltrated cells found in mantle were apparently intact (Figure 8B). The amount of circulating cells infiltrating this tissue was relatively low compared to gills. A number of epithelial cells showed enlarged and damaged nucleus compared to intact cells (arrowheads, Figure 8B).

**Figure 9:**
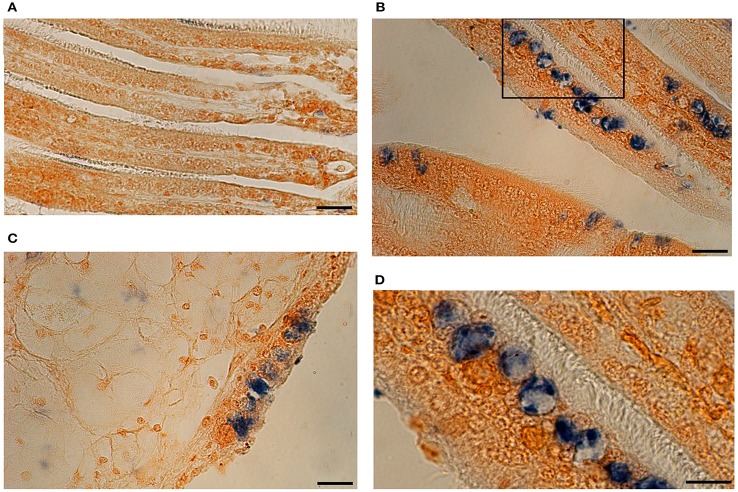
*****In situ*** Hybridization photomicrographs of an uninfected oyster (A)** and a heavily infected individual **(B–D)**. OsHV-1 DNA is marked in blue. **(B)** Gill tissue section with strong blue labeling in epithelia. **(C)** Section showing mantle tissue and epithelial cells. **(D)** Detail of picture **(B)** showing positive marked cells. (Scales bars **A–C**: 20 μm; **D**: 10 μm).

Detection of virus DNA by ISH was characterized by blue precipitates into host cells. Uninfected oyster showed no specific labeling (Figure 9A). Although, a remaining light blue could be observed in some areas this was due to some residual staining and not to positive OsHV-1 marking. In further confirmation of histology observations, blue precipitates were very abundant in gill epithelial cells of infected animals (Figure 9B). The intensity of the signal was also strong in enlarged cells that showed clear blue precipitates in the cytoplasm (Figures 9B,D). Degradation of tissue probably due to the *in situ* hybridization procedure was more evident in mantle (Figure 9C). Cell structures in epithelial cells were not easily distinguished and blue staining was more diffuse occupying mainly enlarged nuclei.

## Discussion

Summer 2013 in Ireland was remarkable for warmer temperatures than average and dry weather, which allowed for a seasonal study at sites with different ranges of sea water temperatures. Indeed, July 2013 was 3.5°C above average and buoys registered the highest sea surface temperature ever recorded in this region (http://www.met.ie/climate/MonthlyWeather/clim-2013-Jul.pdf). The study sites, with different records of oyster mortality and infectivity, were selected on the basis of previous information (Cotter et al., 2010; Lynch et al., 2012; Peeler et al., 2012). Results derived from this study have shown that mortality, prevalence of OsHV-1 μVar and viral load was higher in spat compared to juveniles. Similar observations were previously reported in a survey in Ireland in 2009 and also in other producer countries such as France (Garcia et al., 2011; Peeler et al., 2012). Larvae, spat and juveniles are the most susceptible stages, nevertheless, adults can also be asymptomatically infected (Arzul et al., 2002). Recent studies have identified several immune genes in spat and juveniles that could be involved in defense against the viral infection (Renault et al., 2011; Jouaux et al., 2013; He et al., 2015; Rosani et al., 2015). However, a more effective immune competence might be acquired in later life stages since adult oysters might be able to inhibit viral replication (Olicard et al., 2005; Segarra et al., 2014).

Managements of stocks, environment and oyster source could contribute to the spread of the disease. Among environmental parameters, sea water temperature is considered a risk factor (EFSA, 2010; Renault et al., 2014a). In order to avoid elevated temperatures and air exposure, oyster producers placed the trestles in deep areas. However, the unusually warm temperatures in summer 2013 in Ireland resulted in temperatures exceeding 16°C. Despite the general higher temperatures that could have induced the emergence of the infection in new areas, no positive detection was observed in oysters from the uninfected area. Indeed, strict control measures were taken recently to preserve this area and avoid the spread of OsHV-1 μVar (http://www.fishhealth.ie/FHU/health-surveillance/oyster-herpes-virus-surveillance).

To evaluate the effect of sea water temperature on disease development, we selected 16°C as a threshold for our analyses and periods of 2 weeks prior to samplings (Burge et al., 2006; EFSA, 2010; Pernet et al., 2012; Jenkins et al., 2013; Renault et al., 2014a). On average, the highest temperature per day was two degrees warmer in the southern site. However, mortality and prevalence of infection in juveniles was relatively low at this site. The hatchery origin of juveniles was different between sites and this might explain an intrinsic genetic predisposition to resist or suffer the infection (Sauvage et al., 2009; Dégremont, 2011).

In order to ascertain the effect of different environmental conditions regardless of genetic variance, we placed naïve spat from the virus free area in the two infected sites. Transferred spat experienced massive mortalities in both sites and virus prevalence was high, which demonstrates the elevated susceptibility of this particular stock and the importance of keeping its origin area free of virus. Spat oysters were infected 2 weeks after being transferred in both sites, demonstrating a fast horizontal transmission between oyster stocks (Le Deuff et al., 1994; Schikorski et al., 2011a; Petton et al., 2013; Keeling et al., 2014). However, mortality was detected more rapidly in spat that cohabited with heavy infected juveniles. This might be explained by a faster infectivity progress due to the high prevalence of adjacent stocks.

Spat mortality was associated with prevalence of infection at both sites and also by elevated temperatures at the warmer site, suggesting an important impact of temperature in early stages of life cycle (Malham et al., 2009; Cotter et al., 2010). We observed mortality levels much higher than previous years (Clegg et al., 2014) and we hypothesize that the increase in sea water temperature might have intensified the infection. Moreover, the sudden temperature increase might also directly impact on spat survival.

Quantification of the viral load also revealed higher susceptibility in spat since the amount of virus particles was 10-fold higher than in juveniles. This fact is particularly interesting considering that spat were naïve and were relayed later than juveniles in the field. Moreover, virus DNA amounts in spat were higher than previous levels observed in dead animals after experimental injection and during mortality outbreaks which might reflects the severity of this natural infectivity process (Burge et al., 2011; Martenot et al., 2011; Schikorski et al., 2011a,b; Pernet et al., 2012).

OsHV-1 variants were characterized by regular PCR and partial sequencing of the C region (Renault et al., 2001; Lynch et al., 2013). Nucleotide sequences obtained in three of the four sets of samples perfectly matched with OsHV-1 μVar. The previously described Irish variant (Lynch et al., 2012) was not found in this study although a recent retrospective study detected it in different points of the Irish coast in samples collected between 2008 and 2012 (Morrissey et al., 2015). We also obtained a consensus sequence that differed in one nucleotide deletion compared to OsHV-1 μVar. This modification was found at the left terminus of the fourth coding fragment that comprise the ORF5 (Davison et al., 2005). Although, polymorphisms in this specific area of the C region were previously reported (Martenot et al., 2012; Shimahara et al., 2012; Morrissey et al., 2015) the attention was mainly focused on the ORF4 and the non-coding region containing the microsatellite area (Segarra et al., 2010; Martenot et al., 2011, 2012; Jenkins et al., 2013; Renault et al., 2014b; Bai et al., 2015). However, discrimination of variants attending solely to the microsatellite might ignore the presence of other variants and recent studies also target flanking regions (Mineur et al., 2014). Our results highlight the importance of the ending region of ORF 5 to identify polymorphisms. This part of the genome can be particularly useful to distinguish microvariants especially in the case of equal number of repetitions in the microsatellite area.

Even though there was close proximity between animals, spat relayed to Site B were infected with OsHV-1 μVar and not with the new microvariant that affected juveniles already settled in the same area. This might indicate that the predominant variant in this area is OsHV-1 μVar. Favored by an optimal replication temperature, multiple reservoirs and carriers might be releasing and transmitting infective particles to the environment and naïve animals (Burge et al., 2011; Paul-Pont et al., 2013; Petton et al., 2013). We hypothesized that juveniles might have acquired the infection with the new microvariant before being settled in the culturing area. This infection could remain in a latent state until favorable conditions trigger the replication (Arzul et al., 2002; Sauvage et al., 2009; Peeler et al., 2012).

The ultimate objective of this study was the selection of oysters from the field after a mortality outbreak for future analysis focused on less susceptibility to OsHV-1. In order to corroborate that oysters that survived in the field were indeed less susceptible; we carried out an experimental trial on survivors. We focused on spat animals as this stage is more susceptible. Moreover, the fact that elevated temperatures also contributed to massive mortalities in the warmer site lead us to discard those animals for these analyses. However, spat relayed in the other site experienced mortalities exclusively related to the infection and a set of valuable survivors could be collected after the mortality outbreak. Our strategy to select those animals was based on mass selection. Compared to family selection, this approach has the advantage of being simpler and more similar to husbandry practices and it was postulated as a good option for animal selection (Dégremont et al., 2015b). After being injected with OsHV-1, the naïve stock experienced more losses than the stock of surviving animals previously exposed to the virus during the natural outbreak. These results might demonstrate that these animals are indeed less susceptible to OsHV-1 μVar. To our knowledge this is the first report of selection of oysters after a natural outbreak followed by an experimental injection. Although, the approach for selecting animals and also the method to test susceptibility were not tested before, the survival values at an early stage were similar to those previously observed in the field (Dégremont, 2011; Dégremont et al., 2015a,b).

As a confirmatory method to corroborate a true infection in naturally infected animals, we carried out histology and *in situ* hybridization on heavily infected animals. Histological observation showed the characteristic features of infected cells such as enlargement and chromatin margination (Hine et al., 1992; Renault and Cochennec, 1994; Friedman et al., 2005; Burge et al., 2006). In order to target viral DNA, we tested for the first time DNA probes synthethized with OHVC and OHVD primers (Lynch et al., 2013). These probes labeled mainly viral DNA in gill and mantle cells. This finding agreed with previous studies on histological detection and quantification of virus in different tissues (Arzul et al., 2002; Schikorski et al., 2011a; Jenkins et al., 2013). Although, to a lesser extent, epithelial cells in gills and mantle were also positively marked. In contrast to our findings, a recent study found mainly viral DNA in connective and muscle tissues (Corbeil et al., 2015). The explanation for this difference in results might be found in the process of infection, since these authors utilized intramuscular injection of viral particles to stimulate an experimental infection. In our case, oysters were naturally infected and therefore tissues in direct contact to the environment might be more easily infected. The probes used in this study are shorter compared to those previously used to detect OsHV-1 (Arzul et al., 2002; Lipart and Renault, 2002; Jenkins et al., 2013) and it was described that the smaller probes might give better signals (Moench et al., 1985). Our probes successfully marked viral DNA with a strong signal and could be considered as candidates for routine confirmatory and diagnostic tools.

In conclusion, different diagnosis methods including molecular detection, histological observation and *in situ* hybridization were combined in this study for a better identification of infected stocks during a mortality outbreak. The survey carried out at three points of the Irish coast highlighted the influence of the environment to trigger the infection and also the intrinsic resistance of the oysters to suffer or overcome the disease. The unusual high temperatures intensified the infection process in the field allowing an initial selection of survivors that were subsequently tested under experimental conditions. This approach allowed a successful strategy for selection of less susceptible animals. Future analysis on these selected animals will be carried out to deepen on those traits that might confer less susceptibility against the viral infection.

## Author contributions

MP, SL, and SC: Designed the work; MP, GD, SH, and AO: Collected and processed the samples; MP: Analyzed and interpreted the data; MP, SL, and SC: Wrote, edited and approved the final version of the manuscript.

## Funding

The project HERPISH (Herpes virus in Irish oysters and identification of resistant stocks) was funded by the Seventh EU Framework Programme (FP7- PEOPLE 327932) under the Marie Curie Action.

### Conflict of interest statement

The authors declare that the research was conducted in the absence of any commercial or financial relationships that could be construed as a potential conflict of interest.
